# The effect of bupivacaine on analgesia and safety in patients undergoing hemorrhoidectomy: a meta-analysis

**DOI:** 10.3389/fphar.2023.1331965

**Published:** 2024-05-01

**Authors:** Haixia Lu, Min Cai, Dongxi Zhou, Weiwei Li, Hanzhong Cao

**Affiliations:** ^1^ Medical College of Nantong University, Nantong, China; ^2^ Department of Anesthesiology, Hai’an Hospital of Traditional Chinese Medicine, Hai’an, China; ^3^ Department of Anesthesiology, Funing People’s Hospital of Jiangsu, Yancheng, China

**Keywords:** bupivacaine, hemorrhoidectomy, adverse reactions, meta-analysis, effect and safety

## Abstract

**Aim:** There is no meta-analysis reporting the analgesic effect and safety of bupivacaine in patients undergoing hemorrhoidectomy. This meta-analysis provides quantitative evidence of the effect of bupivacaine in hemorrhoidectomy.

**Methods:** Studies were searched from PubMed, Embase, the Cochrane Library, and the Web of Science. Standardized mean difference (SMD), weighted mean difference (WMD), and odds ratios (ORs) with 95% confidence interval (CI) were used as effect indicators. Heterogeneity was assessed using the *I* ^2^ index, and sensitivity analysis was conducted to determine the effect of the single study on the pooled results.

**Results:** A total of 18 studies were included in this meta-analysis. The pain level at 48 h was lower in the bupivacaine-combined other drug group than in the other drug group (WMD = −0.65, 95% CI: 1.18 to −0.11, and I^2^ = 37.50%). Compared to the bupivacaine group, the odds of pruritus (OR = 12.11, 95% CI: 1.49–98.59, and I^2^ = 0%) and urinary retention (OR = 4.45, 95% CI: 1.12–17.70, and I^2^ = 0%) were higher, and the pain level at 6 h (WMD = −2.13, 95% CI: 3.22 to −1.04, and I^2^ = 64.30%), at 12 h (WMD = −1.55, 95% CI: 2.19 to −0.90, and I^2^ = 56.10%), and at 24 h (SMD = −1.15, 95% CI: 1.89 to −0.42, and I^2^ = 82.5%) were lower in the bupivacaine-combined other drug group.

**Conclusion:** Bupivacaine-combined other drugs had a good analgesic effect after hemorrhoidectomy, but the adverse reactions should be considered.

## Introduction

Hemorrhoids are the most common disease in and around the anus, with clinical symptoms including itching, bleeding, pain, and lumps near the anus (Sandler and Peery, 2019). Grade III or IV hemorrhoids require surgical treatments because they do not respond to pharmacotherapy (Zhang et al., 2020). Hemorrhoidectomy is a common surgery used to treat grade III and IV internal hemorrhoids and extensive external hemorrhoids; however, this surgery may lead to severe postoperative pain (Lohsiriwat and Jitmungngan, 2022). Pain and nausea are two common complications after hemorrhoidectomy, which lead to increased postoperative medication intake, delayed discharge, and frequent hospitalization (Medina-Gallardo et al., 2017). Therefore, pain prevention and management after hemorrhoidectomy is an emphasized problem for patients with hemorrhoids.

Some local anesthetics have been used, but the action duration of the anesthetics still needs to be prolonged to meet patient requirements (Farag and Esmat, 2016). Bupivacaine is a long-acting local anesthetic drug (Farag and Esmat, 2016). Like other local anesthetics, bupivacaine exerts its effect by inhibiting the initiation and conduction of nerve impulses, providing a non-opioid analgesic effect (Chitty et al., 2022). Previous studies have reported the analgesic effect of bupivacaine in patients undergoing hemorrhoidectomy (Hatami et al., 2022; Steen et al., 2022). Hatami et al. (2022) reported that the pain level was lower in the bupivacaine group than in the placebo group at 2 h, 4 h, 8 h, 12 h, and 24 h after hemorrhoidectomy in patients with grade III and IV hemorrhoids. Steen et al. (2022) reported that bupivacaine reduced the pain level of patients with symptomatic hemorrhoids after hemorrhoidectomy. However, there were some limitations in the individual original studies, including insufficient sample size or being limited to one region. A meta-analysis is a powerful tool that can combine the results of two or more individual studies, demonstrates a good evidence advantage, and contributes to healthcare decision making (Mohd-Ali and Chen, 2021; Ozek et al., 2021). Jiang et al. (2023) conducted a meta-analysis and reported the effect of bupivacaine on the postoperative analgesic, rehabilitation, and safety outcomes for surgical wound infiltration. However, there is no meta-analysis reporting the analgesic effect and safety of bupivacaine in patients undergoing hemorrhoidectomy.

Therefore, we performed a systematic review and meta-analysis based on previously published studies, which included a larger sample size on a worldwide basis, to comprehensively explore the effect of bupivacaine on analgesia and safety after hemorrhoidectomy.

## Methods

### Literature search strategy

This meta-analysis was performed according to Preferred Reporting Items for Systematic Reviews and Meta-Analyses (PRISMA) guidelines (Page et al., 2021). Literature search was performed in PubMed, Embase, the Cochrane Library, and the Web of Science up to June 2023. The literature search and screening were performed by two independent researchers (HXL and MC), and dispute was solved by discussion to reach consensus. The search terms are given in Supplementary File S1.

### Inclusion and exclusion criteria

Studies meeting the following criteria were included: 1) patients undergoing hemorrhoidectomy; 2) intervention and control: bupivacaine-combined other drugs vs. other drugs, bupivacaine vs. other drugs, liposomal bupivacaine vs. other drugs, and bupivacaine combined other drugs vs. bupivacaine; 3) outcomes: analgesic effect and safety; and 4) study type: randomized controlled trials and cohort study.

Other drugs included hormones, antibiotics, non-steroidal drugs, and opioid drugs. The analgesic effect was assessed using the immediate pain level, pain level at 6 h, 12 h, 24 h, and 48 h and defecation, pain-free time, and cumulative pain intensity score. Safety was assessed by adverse reactions (nausea, urinary retention, bleeding, vomiting, dyschezia, fever, and pruritus), length of hospital stay, and time to restore daily life.

Studies meeting the following criteria were excluded: 1) animal studies; 2) not published in English; 3) case reports, conference abstracts, guidelines and expert consensus, editorial material, reviews, and meta-analysis; and 4) not relevant to the topic.

### Data extraction and quality appraisal

Two researchers (HXL and MC) independently extracted the following data: the first author, year of publication, country, study design, population, groups, intervention, sample size, gender, age, body mass index (BMI), duration of surgery, number of hemorrhoids, American Society of Anesthesiologists (ASA) classification, and hemorrhoid grade. A third researcher (HZC) provided consultation if conflicts existed.

The quality of cohort studies was assessed using the Newcastle–Ottawa Scale (NOS), which is a 9-point scale and divided the studies into poor (0–3 points), fair (4–6 points), and good quality (7–9 points) (Wells et al., 2011). The RCT quality was assessed using the modified Jadad scale, which is a 7-point scale and divided the studies into poor quality (1–3 points) and good quality (4–7 points) (Jadad et al., 1996; Chen et al., 2020).

### Statistical analysis

For the pain level assessed using different pain scales, the standardized mean difference (SMD) and 95% confidence interval (CI) were estimated. For the pain level assessed using the same pain scale, the weighted mean difference (WMD) and 95% CI were estimated. The odds ratios (ORs) and 95% CI were estimated for the categorical data. Heterogeneity between studies was assessed using the *I* ^2^ index. A random-effects model was used if I^2^ ≥ 50%, and a fixed-effects model was used if I^2^ < 50%. Sensitivity analysis was performed to determine the effect of the individual included study on the pooled results by eliminating the studies one by one. All statistical analyses were performed using STATA (15.1) (StataCorp, College Station, TX, United States).

## Results

### Study selection and characteristics of selected studies

**FIGURE 1:**
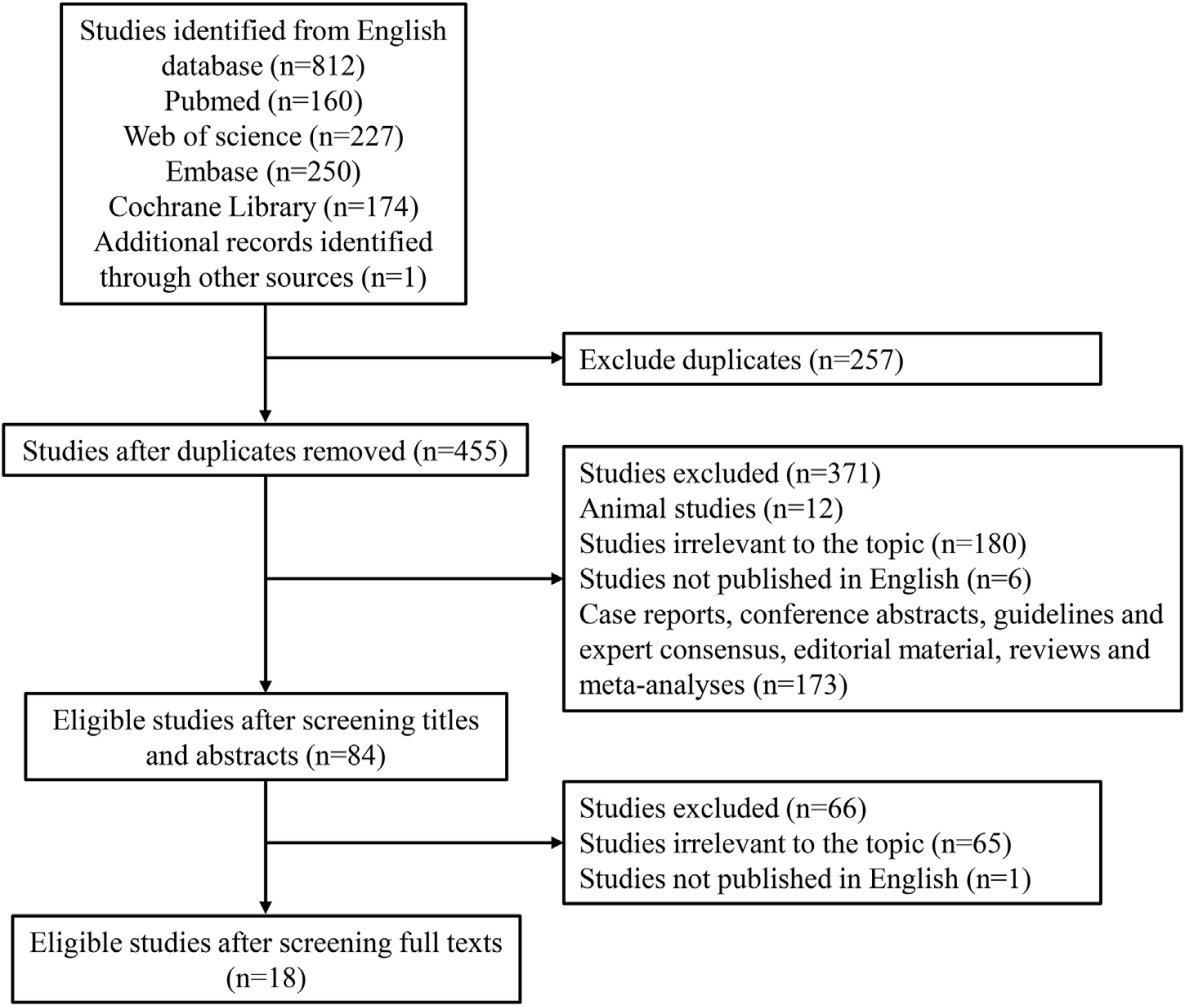
Flowchart of study selection.

Figure 1 demonstrates the search process, which identified a total of 812 citations using the search strategy. Of these, 257 duplicates were excluded. After screening abstracts or titles, 12 animal studies, 180 irrelevant studies, 6 non-English studies, and 173 other study types (case reports, conference abstracts, guidelines and expert consensus, editorial material, reviews, and meta-analyses) were excluded. Furthermore, 65 irrelevant studies and 1 non-English study were excluded based on full-text reading. Finally, 18 eligible studies were included in our meta-analysis (Jirasiritham et al., 2004; Naja et al., 2005; Ng et al., 2006; Imbelloni et al., 2007; Gorfine et al., 2011; Haas et al., 2012; Rajabi et al., 2012; Moreira et al., 2014; Sim and Tan, 2014; Farag and Esmat, 2016; Shashikala and Prathibha, 2016; Ruiz-Castro et al., 2017; Chitty et al., 2022; Cui et al., 2022; Hatami et al., 2022; Medina-Gallardo et al., 2022; Steen et al., 2022; Shahrokhzadeh et al., 2023). The characteristics of the 18 eligible studies are shown in Table 1. There were 17 randomized controlled trials and 1 cohort study. Two studies were assessed to be of low quality, and 16 studies were assessed to be of high quality.

**TABLE 1:** Characteristics of the included studies.

Author	Year	Country	Study design	Population	Group	Intervention	N	Male/female	Age (year)
Shahrokhzadeh	2023	Iran	RCT	Patients with hemorrhoidectomy	Bupivacaine + ketorolac	4 mL 0.5% Marcaine + 1 mL ketorolac at the surgical site	28	14/14	29.89 ± 8.80
					Bupivacaine + ketorolac	4 mL 0.5% Marcaine at the surgical site + 1 mL ketorolac intramuscularly	28	13/15	39.32 ± 8.46
					Bupivacaine	4 mL 0.5% Marcaine at the surgical site	28	12/16	30.93 ± 8.33
Steen	2022	Australia	RCT	Patients with hemorrhoidectomy	Bupivacaine + adrenaline	10 mL of bupivacaine 0.5% with 1:200,000 adrenaline	39	14/25	48
					Control	Standard postoperative oral analgesia regimen	40	14/26	45
Medina	2022	Spain	RCT	Patients with hemorrhoidectomy	Bupivacaine + triamcinolone acetonide	Infiltrated in the surgical wound with a single dose of 1 mL of triamcinolone acetonide (40 mg) + 9 mL of bupivacaine hydrochloride 0.50% (45 mg)	64	24/40	49.7 (47.8–53.9)**
					Blank	Not receiving any intervention	64	31/33	50.8 (46.7–52.4)**
Hatami	2022	Iran	RCT	Patients with hemorrhoidectomy	Bupivacaine	Perianal infiltration of 0.25% bupivacaine, (overall volume of injections was approximately 3 cc in each site)	30	NA	18–75 (range)
					Tramadol	Perianal infiltration of tramadol (2 mg/kg) (overall volume of injections was approximately 3 cc in each site)	30	NA	18–75 (range)
					Saline	Perianal infiltration of normal saline (as placebo) (overall volume of injections was approximately 3 cc in each site)	30	NA	18–75 (range)
Ruiz	2017	Spain	RCT	Patients with hemorrhoidectomy	Bupivacaine + morphine	3 mg of hyperbaric bupivacaine 0.5% Mini-Plasco^®^ BraUn 0.6 mL with 50 μg (mcg) of morphine hydrochloride 0.1% (MH) Serra^®^ diluted with 1.4 mL normal saline 0.9%, up to a total volume of 2 mL	30	17/13	45.8 ± 10.5
					Bupivacaine	5 mg of hyperbaric bupivacaine 0.5% Mini-Plasco^®^ BraUn 1 mL, diluted with normal saline 0.9% 1 mL, up to a total volume of 2 mL	33	20/13	51.3 ± 10.8
Shashikala	2016	Mysore	RCT	Patients with hemorrhoidectomy	Bupivacaine + dexmedetomidine	Dexmedetomidine [diluting 1 mL (containing 100 µg) to 5 mL with normal saline]; 0.5 mL of this diluted solution was taken using an insulin syringe and added to the syringe containing 1 mL of 0.5% hyperbaric bupivacaine	30	NA	18–50 (range)
					Bupivacaine	0.5 mL of normal saline taken in an insulin syringe added to 1 mL of 0.5% hyperbaric bupivacaine	30	NA	18–50 (range)
Farag	2016	Egypt	RCT	Patients with hemorrhoidectomy	Bupivacaine	Injected 20 mL of 0.25% plain bupivacaine in caudal epidural space	30	13/17	30.2 ± 5.7
					Tramadol	Injected 1 mg/kg tramadol in 20 mL normal saline	30	14/16	27.7 ± 4.9
					Tramadol	Injected 2 mg/kg tramadol in 20 mL normal saline	30	12/18	28 ± 5.2
Sim	2014	Singapore	RCT	Patients with hemorrhoidectomy	Bupivacaine + methylene blue	Intradermal injection of 4 mL 1% methylene blue and 16 mL 0.5% Marcaine	37	16/21	42.3 ± 10.0
					Bupivacaine	16 mL 0.5% Marcaine and 4 mL saline without methylene blue	30	20/10	45.2 ± 12.3
Moreira	2014	Brazil	RCT	Patients with hemorrhoidectomy	Bupivacaine + morphine	Spinal subarachnoid block with 7 mg of heavy bupivacaine and 80 µg of morphine (0.2 mg/mL)	17	5/12	40.5 ± 12.6
					Bupivacaine	Spinal anesthesia with 7 mg of heavy bupivacaine in distilled water	18	10/8	46.1 ± 14.3
Rajabi	2012	Iran	RCT	Patients with hemorrhoidectomy	Bupivacaine	Preoperative ischiorectal block with bupivacaine 0.25%	30	15/15	NA
					Saline	Preoperative ischiorectal block with normal saline	30	16/14	NA
Imbelloni	2007	Brazil	RCT	Patients with hemorrhoidectomy	Bupivacaine	The bilateral pudendal block with 20 mL of> 0.25% bupivacaine on each side	50	24/26	44.9 ± 10.5
					Ketoprofen	Medicated with 100 mg ketoprofen in 100 mL of lactate ringer solution	50	28/22	43.1 ± 11.9
Ng	2006	China	RCT	Patients with hemorrhoidectomy	Bupivacaine	Infiltration of 10 mL of 0.5% bupivacaine to the surgical area before skin incision	27	12/15	48.3 ± 10.3
					Oral metronidazole	400 mg oral metronidazole three times daily for 7 days after the operation	26	9/17	52 ± 16
					Bupivacaine + oral metronidazole	Infiltration of 10 mL of 0.5% bupivacaine to the surgical area before skin incision + 400 mg oral metronidazole three times daily for 7 days after the operation	26	12/14	48.7 ± 12.1
					Control	Control group	26	7/19	46.7 ± 12.4
Naja	2005	Sweden	RCT	Patients with hemorrhoidectomy	Bupivacaine + lidocaine + fentanyl + clonidine	Pudendal injection of a local anesthetic mixture (bupivacaine, lidocaine, fentanyl and clonidine), 0.7 mL kg	30	18/12	37.6 ± 9.8
					Saline	A pudendal injection of normal saline	30	21/9	37.0 ± 10.2
Jirasiritham	2004	Thailand	RCT	Patients with hemorrhoidectomy	Bupivacaine	0.5% bupivacaine 1–3 mL around the base of each hemorrhoid 10 min before operation	72	43/29	37.48 + 13.63
					Blank	Control group	70	31/39	40.45 + 13.03
Chitty	2022	United States	Cohort	Patients with hemorrhoidectomy	Liposomal bupivacaine	Liposomal bupivacaine (10 mL saline mixed with 266 mg/20 mL of Exparel^®^) infiltrated in a fan-like fashion around the perianal tissue	47	22/25	53 (42–62)**
					Bupivacaine	0.25% bupivacaine (125 mg/50 mL of Marcaine^®^) infiltrated in a fan-like fashion around the perianal tissue	47	17/30	49 (40–60)**
Cui	2022	China	RCT	Patients with hemorrhoidectomy	Sustained-release formulation of bupivacaine	HYR-PB21(150 mg) administered in 30 mL and infiltrated into the perianal tissue in a fan-like fashion around the anus	24	13/11	42 (23–68)*
					Sustained-release formulation of bupivacaine	HYR-PB21(300 mg) administered in 30 mL and infiltrated into the perianal tissue in a fan-like fashion around the anus	24	10/14	43 (24–63)*
					Bupivacaine HCl	Bupivacaine HCl administered in 30 mL and infiltrated into the perianal tissue in a fan-like fashion around the anus	23	12/11	41 (26–65)*
Haas	2012	United States	RCT	Patients with hemorrhoidectomy	Liposomal bupivacaine	LB (66 mg) administered via local infiltration	24	17/7	42 ± 11
					Liposomal bupivacaine	LB (199 mg) administered via local infiltration	25	16/9	42 ± 11
					Liposomal bupivacaine	LB (266 mg) administered via local infiltration	25	22/3	46 ± 11
					Bupivacaine HCl	Bupivacaine HCl administered via local infiltration	26	15/11	44 ± 11
Gorfine	2011	United States	RCT	Patients with hemorrhoidectomy	Liposomal bupivacaine	300 mg/30 mL DepoFoam bupivacaine injected at the end of surgery in 5-mL increments, infiltrating the perianal tissues in a fan-like fashion around the anus	95	63/32	48.0 ± 12.2
					Saline	Placebo (0.9% sodium chloride 30 mL) injected at the end of surgery in 5-mL increments, infiltrating the perianal tissues in a fan-like fashion around the anus	94	67/27	48.7 ± 11.9

BMI (kg/m^2^)	Weight (kg)	Height (cm)	Duration of surgery (min)	Number of hemorrhoids	ASA classification	Hemorrhoids grade	Outcomes	QA
26.9 ± 3.6	NA	NA	NA	NA	I and II	2	NPRS	4
26.3 ± 2.4	NA	NA	NA	NA	I and II	2		
25.9 ± 1.9	NA	NA	NA	NA	I and II	2		
NA	NA	NA	31	2.18	NA	NA	Adverse events	6
NA	NA	NA	36	2.33	NA	NA		
NA	NA	NA	NA	NA	I: 19 (29.7%); III: 45 (70.3%)	III: 47; IV: 17	VAS, pain during defecation, postoperative complications	7
NA	NA	NA	NA	NA	I: 21 (32.8%); III: 43 (67.2%)	III: 39; IV: 25		
<30	NA	NA	NA	NA	NA	III and IV	Pain score (VAS)	5
<30	NA	NA	NA	NA	NA	III and IV		
<30	NA	NA	NA	NA	NA	III and IV		
25.1 ± 3.3	NA	NA	28.1 ± 11.1	1: 8 (28.5%); 2: 12 (42.8%); 3: 8 (28.5%)	I: 7 (23.3%); II: 22 (73.3%); III: 1 (3.3%)	NA	Adverse events	7
27.2 ± 4.0	NA	NA	20.5 ± 9.2	1: 12 (36.3%); 2: 13 (39.3%); 3: 8 (24.2%)	I: 8 (24.2%); II: 25 (75.7%); III: 0 (0%)	NA		
NA	NA	NA	NA	NA	I and II	NA	Duration of analgesia	3
NA	NA	NA	NA	NA	I and II	NA		
NA	67.4 ± 7	NA	44.07 ± 6.2	NA	I and II	NA	Duration of analgesia	5
NA	65.4 ± 6.5	NA	43.9 ± 5.9	NA	I and II	NA		
NA	63.8 ± 7.2	NA	4 0.1 ± 5.3	NA	I and II	NA		
NA	NA	NA	20.0 ± 10.9	2.6 ± 0.8	NA	III and IV	Complication, mean postoperative pain scores	7
NA	NA	NA	20.8 ± 10.6	2.6 ± 0.8	NA	III and IV		
NA	71.7 ± 13.2	NA	NA	≥ 3	I: 12 (70.6%); II: 4 (23.5%); III: 1 (5.9%)	III and IV	Postoperative pain	4
NA	69.1 ± 11.7	NA	NA	≥ 3	I: 12 (66.7%); II: 6 (33.3%); III: 0 (0%)	III and IV		
NA	NA	NA	NA	NA	NA	NA	Postoperative pain	4
NA	NA	NA	NA	NA	NA	NA		
24.9 ± 3.2	70.6 ± 13.2	167.8 ± 8.4	NA	NA	I and II	NA	Pain-free period, degree of pain, frequency of codeine analgesic doses, complications	4
25.1 ± 4.6	71.3 ± 14	166.9 ± 8.6	NA	NA	I and II	NA		
NA	NA	NA	NA	NA	NA	III and IV	Pain scores (VAS), analgesic requirements, hospital stay, and recovery	4
NA	NA	NA	NA	NA	NA	III and IV		
NA	NA	NA	NA	NA	NA	III and IV		
NA	NA	NA	NA	NA	NA	III and IV		
NA	70.4 ± 14.0	167.3 ± 7.8	66 ± 18.6	NA	NA	II: 5; III: 11; IV: 14	Pain scores (VAS), adverse events	4
NA	74.5 ± 14.3	168.6 ± 10.4	60 ± 18.6	NA	NA	II: 6; III: 12; IV: 12		
NA	58.80 + 9.76	NA	NA	NA	II and III	NA	Pain-free period, pain severity	3
NA	59.77 + 11.19	NA	NA	NA	II and III	NA		
28.9 (24.6–33.2)**	NA	NA	NA	25 [1: 7 (28%); 2: 16 (64%); 3: 2 (8%)]	II (II–III) **	NA	Pain score (NRS), adverse events	8
28.8 (24.6–34.6) **	NA	NA	NA	28 [1: 8 (28.5%); 2: 19 (67.8%); 3: 1 (3.7%)]	II (II–III) **	NA		
24.9 ± 3.0	71 ± 11	166 ± 9	NA	NA	I–III	NA	Cumulative pain intensity score, adverse event	4
23.5 ± 3.1	65 ± 13	169 ± 7	NA	NA	I–III	NA		
23.6 ± 2.7	66 ± 12	168 ± 10	NA	NA	I–III	NA		
NA	85 ± 25	173 ± 11	NA	2 and 3	I–II: 18 (75%); III–IV: 4 (17%)	NA	Adverse event	5
NA	77 ± 19	171 ± 9	NA	2 and 3	I–II: 23 (92%); III–IV: 2 (8%)	NA		
NA	82 ± 16	174 ± 6	NA	2 and 3	I–II: 22 (88%); III–IV: 2 (8%)	NA		
NA	80 ± 16	171 ± 10	NA	2 and 3	I–II: 19 (73%); III–IV: 2 (8%)	NA		
25.5 ± 3.9	76.3 ± 15.0	172.3 ± 8.5	NA	2 and 3	I: 57 (60%); II: 36 (37.9%); III: 2 (2.1%)	NA	Cumulative pain intensity score, adverse events	4
25.9 ± 3.9	78.7 ± 13.4	174.2 ± 9.4	NA	2 and 3	I: 49 (52.1%); II: 42 (44.7%); III: 3 (3.2%)	NA		

*, range; **, median (IQR); BMI, body mass index; ASA, American Society of Anesthesiologists; QA, quality assessment; RCT, randomized controlled trial; HYR-PB21, sustained-release formulation of bupivacaine; HCI, hydrochloric acid; NA, not applicable; NPRS, numerical pain rating scale; VAS, visual analog scale; NRS, numeric rating scale.

### Effect of bupivacaine on analgesia and safety after hemorrhoidectomy

**FIGURE 2:**
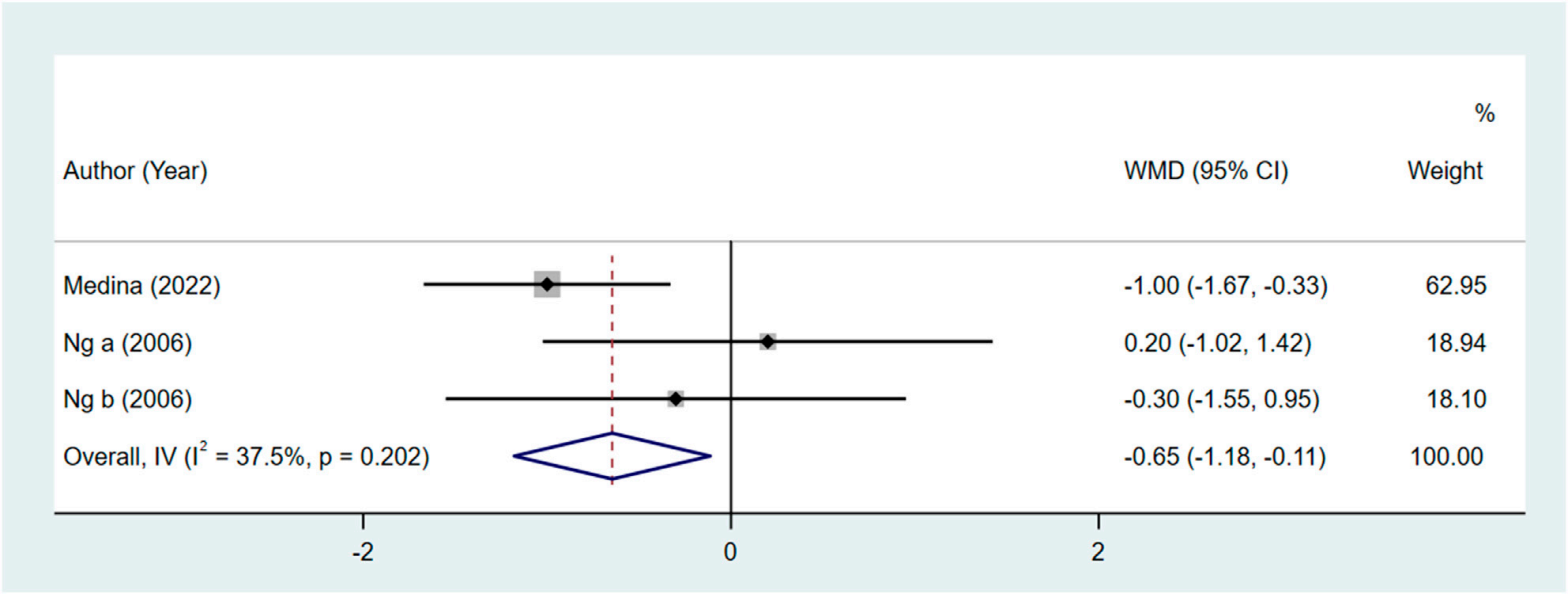
Forest plot of the pain level at 48 h between the bupivacaine-combined other drug group and other drug group.

Comparing the bupivacaine-combined other drug group with the other drug group, the pain level at 48 h in the bupivacaine-combined other drug group was lower than in the other drug group, with the pooled WMD of −0.65 (95% CI: −1.18 to −0.11, I^2^ = 37.50%) (Figure 2). There was no significant difference in the pain level immediately, at 24 h, and at defecation, time to restore daily life, and urinary retention between the bupivacaine-combined other drug group and the other drug group (all *p* > 0.05).

**FIGURE 3:**
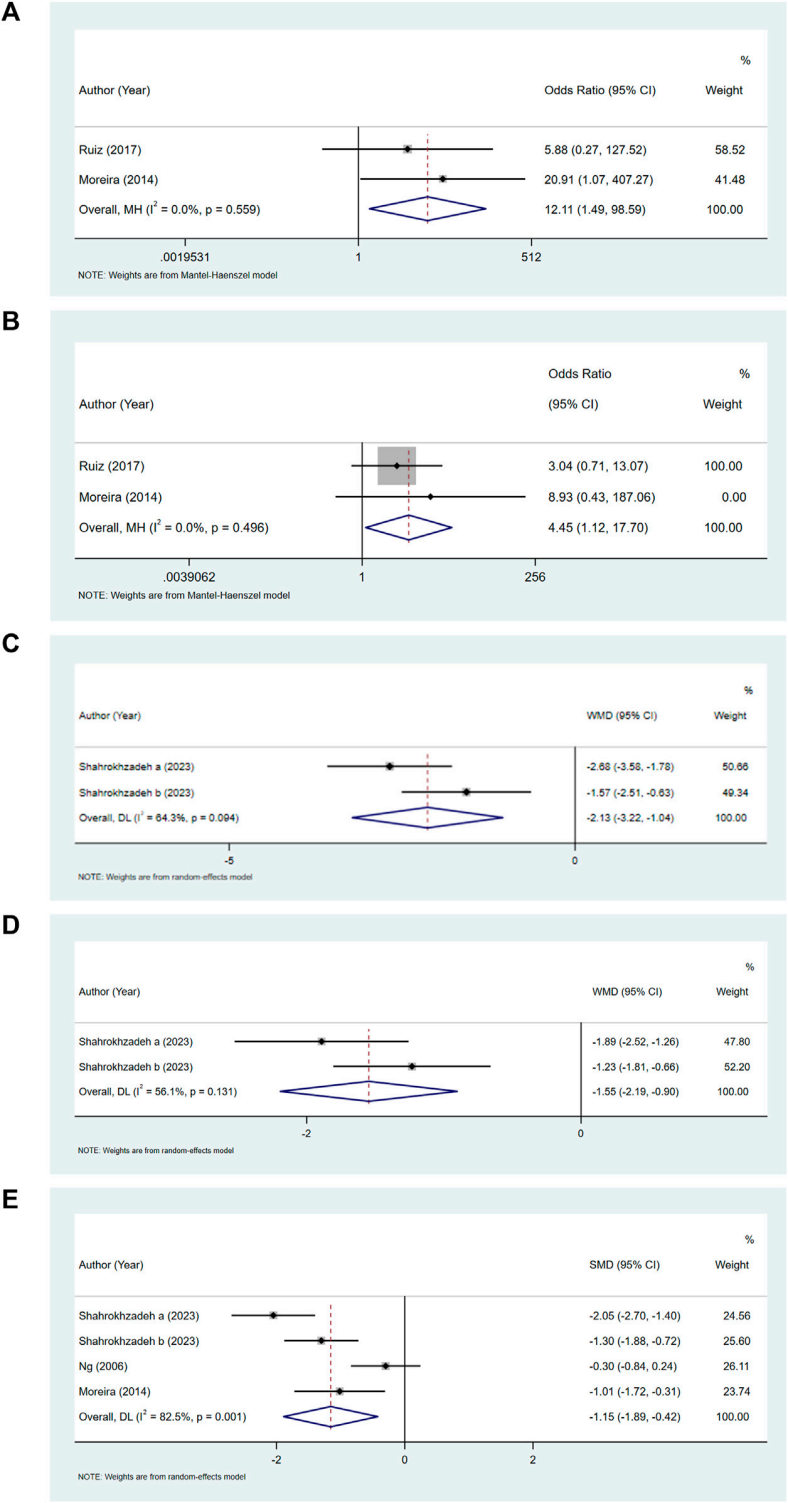
Forest plot of pruritus **(A)**, urinary retention **(B)**, and pain level at 6 h **(C)**, 12 h **(D)**, and 24 h **(E)**.

Comparing the bupivacaine-combined other drug group with the bupivacaine group, we found that the odds of pruritus (OR = 12.11, 95% CI: 1.49–98.59, and I^2^ = 0%) (Figure 3A) and urinary retention (OR = 4.45, 95% CI: 1.12–17.70, and I^2^ = 0%) (Figure 3B) were higher in the bupivacaine-combined other drug group than in the bupivacaine group. The pain level at 6 h (WMD = −2.13, 95% CI: 3.22 to −1.04, and I^2^ = 64.30%) (Figure 3C) and 12 h (WMD = −1.55, 95% CI: 2.19 to −0.90, and I^2^ = 56.10%) (Figure 3D) was found to be lower in the combination group than in the bupivacaine group. The pooled results also showed that the pain level at 24 h was lower in the bupivacaine-combined other drug group than in the bupivacaine group (SMD = −1.15, 95% CI: −1.89 to −0.42, and I^2^ = 82.5%) (Figure 3E).

Comparing the bupivacaine group with other drug group, no significance was observed in the pain level at 12 h, at 24 h, and at defecation and pain-free time between the two groups (all *p* > 0.05).

Comparing the liposomal bupivacaine group with other drugs, we also observed no significance in the cumulative pain intensity score, vomiting, dyschezia, and fever between the two groups (all *p* > 0.05).

### Publication bias and sensitivity analysis

Sensitivity analysis was carried out via eliminating the studies one by one to determine the effect of the individual included study on the pooled results. The results displayed that the pooled results were not affected by omitting the individual study (Table 2). Publication bias was tested if more than nine studies were included for one outcome (Sterne et al., 2011). In this meta-analysis, there were no more than nine studies combined for analysis under one outcome; therefore, publication bias was not assessed.

**TABLE 2:** Meta-analysis results of effect of bupivacaine on analgesia and safety after hemorrhoidectomy.

Outcomes	Number of studies	Sample size	SMD/WMD/OR (95% CI)	*P*	I^2^^(%)^
Bupivacaine-combined other drugs vs. other drugs					
Immediate pain level	1	104	1.05 (−0.01, 2.11)^b^	0.051	0.00
Sensitivity analysis			1.05 (−0.01, 2.11)		
Pain level at 24 h	2	232	−0.39 (−2.04, 1.27)^b^	0.645	83.50
Sensitivity analysis			−0.39 (−2.04, 1.27)		
Pain level at 48 h	2	232	−0.65 (−1.18, −0.11)^b^	0.017	37.50
Sensitivity analysis			−0.65 (−1.18, −0.11)		
Pain level at defecation	3	292	−0.85 (−1.85, 0.15)^a^	0.094	93.30
Sensitivity analysis			−0.85 (−1.85, 0.15)		
Time to restore daily life	2	164	−0.33 (−1.39, 0.73)^b^	0.540	90.90
Sensitivity analysis			−0.33 (−1.39, 0.73)		
Urinary retention	2	139	0.13 (0.02, 1.10)^c^	0.062	0.00
Sensitivity analysis			0.13 (0.02, 1.10)		
Bupivacaine vs. other drugs					
Pain level at 12 h	3	280	−1.09 (−2.62, 0.44)^a^	0.162	96.80
Sensitivity analysis			−1.09 (−2.62, 0.44)		
Pain level at 24 h	3	325	−0.36 (−1.09, 0.37)^a^	0.33	90.10
Sensitivity analysis			−0.36 (−1.09, 0.37)		
Pain level at defecation	2	226	0.13 (−0.42, 0.68)^a^	0.649	77.00
Sensitivity analysis			0.13 (−0.42, 0.68)		
Pain-free time	3	360	−2.43 (−7.25, 2.39)^b^	0.324	99.20
Sensitivity analysis			−2.43 (−7.25, 2.39)		
Liposomal bupivacaine vs. other drugs					
Cumulative pain intensity score	2	281	−2.40 (−5.55, 0.74)^b^	0.134	98.70
Sensitivity analysis			−2.40 (−5.55, 0.74)		
Vomiting	3	432	0.52 (0.22, 1.27)^c^	0.154	0.00
Sensitivity analysis			0.52 (0.22, 1.27)		
Dyschezia	3	432	0.46 (0.21, 1.03)^c^	0.058	0.00
Sensitivity analysis			0.46 (0.21, 1.03)		
Fever	2	281	1.99 (0.49, 8.12)^c^	0.335	0.00
Sensitivity analysis			1.99 (0.49, 8.12)		
Bupivacaine-combined other drugs vs. bupivacaine					
Pruritus	2	98	12.11 (1.49, 98.59)^c^	0.020	0.00
Sensitivity analysis			12.11 (1.49, 98.59)		
Urinary retention	2	98	4.45 (1.12, 17.70)^c^	0.034	0.00
Sensitivity analysis			4.45 (1.12, 17.70)		
Nausea and vomiting	2	98	0.69 (0.10, 4.56)^c^	0.7	0.00
Sensitivity analysis			0.69 (0.10, 4.56)		
Immediate pain level	2	165	−0.57 (−1.52, 0.38)^a^	0.242	88.70
Sensitivity analysis			−0.57 (−1.52, 0.38)		
Pain level at 6 h	1	112	−2.13 (−3.22, −1.04)^b^	< 0.001	64.30
Sensitivity analysis			−2.13 (−3.22, −1.04)		
Pain level at 12 h	1	112	−1.55 (−2.19, −0.90)^b^	< 0.001	56.10
Sensitivity analysis			−1.55 (−2.19, −0.90)		
Pain level at 24 h	3	200	−1.15 (−1.89, −0.42)^a^	0.002	82.5
Sensitivity analysis			−1.15 (−1.89, −0.42)		

^a^ SMD, standardized mean difference.

^b^ WMD, weighted mean difference.

^c^ OR, odds ratio; CI, confidence interval.

## Discussion

Bupivacaine is a long-term local anesthetic drug that provides non-opioid analgesic effects by inhibiting the initiation and transmission of nerve impulses (Farag and Esmat, 2016; Chitty et al., 2022). Previous studies have reported the analgesic effect and safety of bupivacaine in patients undergoing hemorrhoidectomy (Hatami et al., 2022; Steen et al., 2022), while some limitations existed in the individual original studies, such as smaller sample size or performed in only one region. The meta-analysis could combine the results of two or more individual studies and demonstrate a good evidence advantage. This meta-analysis provided quantitative evidence of the effect of bupivacaine in hemorrhoidectomy. According to the analysis of our study, compared to the other drug group, the bupivacaine-combined other drug group induced a significant reduction in the postoperative pain level at 48 h. Compared to the bupivacaine group, the bupivacaine-combined other drug group decreased the postoperative pain level at 6 h, 12 h, and 24 h while increasing the odds of pruritus and urinary retention. In general, these findings confirmed the analgesic effects of bupivacaine-combined other drugs in hemorrhoidectomy, but the adverse reactions should be considered.

Postoperative pain remains a significant problem in hemorrhoidectomy because up to 40% of patients experience severe pain (Medina-Gallardo et al., 2022). Preemptive analgesia is a simple method to decrease the level and duration of postoperative pain (Van Backer et al., 2018). It is believed that preoperative blockade of the pain pathway can decrease the amount and duration of postoperative pain perception by preventing nociceptive input from afferent stimuli to the central nervous system during the surgical process (Van Backer et al., 2018). A study has shown that preemptive analgesia with perianal infiltration of 0.5% bupivacaine can better alleviate pain after hemorrhoidectomy (Jirasiritham et al., 2004). Medina et al. found that bupivacaine-combined triamcinolone acetonide achieved better post-hemorrhoidectomy pain relief at 48 h than the blank group (Medina-Gallardo et al., 2022). Accordingly, the results of our meta-analysis showed a significant pain reduction at 48 h in the bupivacaine-combined other drug group than in the other drug group. In addition, the present meta-analysis found that the bupivacaine-combined other drug group had a lower postoperative pain level at 6 h, 12 h, and 24 h compared to the bupivacaine group. In the included studies reporting these three outcomes, the other drugs were defined as ketorolac, morphine, and metronidazole (Ng et al., 2006; Moreira et al., 2014; Shahrokhzadeh et al., 2023). The reason for our finding may be that a combination with other drugs may strengthen the initial anesthetic effect of bupivacaine, thereby providing longer postoperative pain control (Ng et al., 2006; Moreira et al., 2014; Shahrokhzadeh et al., 2023).

The main objective of postoperative pain management is to provide sufficient pain relief while reducing adverse reactions (Wick et al., 2017). In this meta-analysis, we found that the bupivacaine-combined other drug group had a higher odds of pruritus and urinary retention. Due to different factors such as temporary detrusor muscle dysfunction, urethral spasm caused by anal pain, and excessive preoperative and postoperative intravenous infusion, urinary retention may occur after hemorrhoidectomy (Salvati and Kleckner, 1957; Cataldo and Senagore, 1991; Zaheer et al., 1998; Toyonaga et al., 2006). In the included studies reporting these two outcomes, the other drug used was morphine (Moreira et al., 2014; Ruiz-Castro et al., 2017). Morphine may cause several adverse reactions, such as pruritus and urinary retention (Moreira et al., 2014; Ruiz-Castro et al., 2017). Our findings suggested that adverse reactions should be considered when using bupivacaine-combined morphine, and whether the benefit of pain control is worth the risk of urinary retention and cutaneous pruritus should be discussed. In the future, more studies are needed to further explore the effect of bupivacaine-combined other drugs on adverse reactions.

This meta-analysis explores the effect of bupivacaine on the analgesia and safety in patients undergoing hemorrhoidectomy. Most of the included original studies are of high quality, and the pooled results show the good analgesic effects of bupivacaine-combined other drugs, which may provide evidence for the extra use of bupivacaine in hemorrhoidectomy for postoperative pain management. Furthermore, there are some limitations to this meta-analysis. First, we only included studies published in English, which may result in language bias. Second, the heterogeneity is high in some results. The dosage and use methods of bupivacaine, as well as the differences in control drugs, may be the source of heterogeneity. Due to the limitations in the included studies, we were unable to further conduct subgroup analysis to explore the sources of heterogeneity. Third, the number of studies is relatively small in some outcomes, which may affect the robustness of the results.

## Conclusion

The results indicated that bupivacaine-combined other drugs had a good effect on pain relief after hemorrhoidectomy, but the adverse reactions should be further discussed.

## Data Availability

The raw data supporting the conclusions of this article will be made available by the authors, without undue reservation.
