# Supplementary material for: The effect of bupivacaine on analgesia and safety in patients undergoing hemorrhoidectomy: a meta-analysis
Source: Front Pharmacol. 2024 May 1;14:1331965. doi: 10.3389/fphar.2023.1331965 (PMC11094319; doi:10.3389/fphar.2023.1331965)
Supplement: Supplementary file 1 [file Table1.DOCX]

Search terms were shown as the following: “Bupivacaine” OR “1-Butyl-N-(2,6-dimethylphenyl)-2-piperidinecarboxamide” OR “Marcain” OR “Bupivacain Janapharm” OR “Bupivacain-RPR” OR “Bupivacain RPR” OR “Bupivacaina Braun” OR “Carbostesin” OR “Bupivacaine Carbonate” OR “Bupivacaine Hydrochloride” OR “Bupivacaine Monohydrochloride, Monohydrate” OR “Buvacaina” OR “Dolanaest” OR “Sensorcaine” OR “Svedocain Sin Vasoconstr” OR “Marcaine” OR “Bupivacaine Anhydrous” AND “Hemorrhoidectomy” OR “Hemorrhoidectomies” OR “Hemorrhoids” OR “Hemorrhoid” OR “Fissure in Ano” OR “Anal Fissure” OR “Fissure, Anal” OR “Anal Ulcer” OR “Anal Ulcers” OR “Ulcer, Anal” OR “Ulcers, Anal” OR “Fistula” OR “Fistulas” OR “Sphincterolysis” OR “Partial resection of internal sphincter” OR “Lateral resection of sphincter” OR “Anal stretch” OR “Balloon dilation” OR “Wound Closure Techniques” OR “Closure Technique, Wound” OR “Closure Techniques, Wound” OR “Technique, Wound Closure” OR “Techniques, Wound Closure” OR “Wound Closure Technique” OR “Surgical Closure Techniques” OR “Closure Technique, Surgical” OR “Closure Techniques, Surgical” OR “Surgical Closure Technique” OR “Technique, Surgical Closure” OR “Techniques, Surgical Closure” OR “Perineoplasty” OR “Fissurectomy” OR “Sphincterotomy” OR “Sphincterotomies” OR “Fistulectomy” OR “Anus Diseases” OR “Anus Disease” OR “Disease, Anus” OR “Diseases, Anus” OR “Anus Neoplasms” OR “Neoplasms, Anal” OR “Anal Neoplasms” OR “Anal Neoplasm” OR “Neoplasm, Anal” OR “Neoplasms, Anus” OR “Anus Neoplasm” OR “Neoplasm, Anus” OR “Anal Cancer” OR “Anal Cancers” OR “Cancer, Anal” OR “Cancers, Anal” OR “Cancer of the Anus” OR “Cancer of Anus” OR “Anus Cancer” OR “Anus Cancers” OR “Rectal Neoplasms” OR “Neoplasm, Rectal” OR “Rectal Neoplasm” OR “Rectum Neoplasms” OR “Neoplasm, Rectum” OR “Rectum Neoplasm” OR “Rectal Tumors” OR “Rectal Tumor” OR “Tumor, Rectal” OR “Neoplasms, Rectal” OR “Cancer of Rectum” OR “Rectum Cancers” OR “Rectal Cancer” OR “Cancer, Rectal” OR “Rectal Cancers” OR “Rectum Cancer” OR “Cancer, Rectum” OR “Cancer of the Rectum” OR “Proctitis” OR “Proctitides” OR “Rectal Prolapse” OR “Prolapse, Rectal” OR “Prolapses, Rectal” OR “Rectal Prolapses” OR “Anus Prolapse” OR “Anus Prolapses” OR “Prolapse, Anus” OR “Rectocele” OR “Rectoceles” OR “Proctocele” OR “Proctoceles” OR “Fecal Incontinence” OR “Incontinence, Fecal” OR “Bowel Incontinence” OR “Incontinence, Bowel” OR “Fecal Soiling” OR “Soilings, Fecal” OR “Rectal Diseases” OR “Rectal Disease” OR “Rectal Disorders” OR “Rectal Disorder” OR “Anorectal Diseases” OR “Anorectal Disease” OR “Anorectal Disorders” OR “Anorectal Disorder”.
